# Transperineal Laser Ablation Treatment for Lower Urinary Tract Symptoms Due to Benign Prostatic Obstruction: Protocol for a Prospective In Vivo Pilot Study

**DOI:** 10.2196/15687

**Published:** 2020-01-21

**Authors:** Rob A A van Kollenburg, Luigi A M J G van Riel, Paul R Bloemen, Jorg R Oddens, Theo M de Reijke, Harrie P Beerlage, Daniel Martijn de Bruin

**Affiliations:** 1 Department of Urology Amsterdam University Medical Centers University of Amsterdam Amsterdam Netherlands; 2 Department of Biomedical Engineering & Physics Amsterdam University Medical Centers University of Amsterdam Amsterdam Netherlands

**Keywords:** transperineal laser ablation, prostatic hyperplasia, lower urinary tract symptoms, laser therapy

## Abstract

**Background:**

Standard surgical treatments for lower urinary tract symptoms (LUTS) due to benign prostatic obstruction (BPO) use a transurethral approach. Drawbacks are the need for general or spinal anesthesia and complications such as hematuria, strictures, and cloth retention. Therefore, a minimal invasive technique under local anesthesia is desired to improve patient safety. Recently, SoracteLite transperineal laser ablation (TPLA) has been introduced as a novel minimal invasive treatment for BPO. The system used is unique because 4 laser sources are independently available. This 1064-nm diode laser induces coagulative necrosis. Moreover, TPLA is unique because it has a transperineal approach and can be performed under local anesthesia in an outpatient setting.

**Objective:**

The primary objective of this study is to determine the safety and feasibility of TPLA treatment for men, who are fit for standard surgery, with LUTS due to BPO. The secondary objectives are to determine functional outcomes by flowmetry and patient-reported outcome measures (PROMs), side effects, and tissue changes observed on imaging.

**Methods:**

This study is a prospective, single center, interventional pilot study IDEAL framework stage 2a and will include 20 patients. Eligible patients are men ≥40 years of age, with a prostate volume of 30 to 120 cc, have urodynamically proven bladder outlet obstruction, and have a peak urinary flow of 5 to 15 mL per second. All patients will undergo TPLA of their prostate under local anesthesia by using the EchoLaser system. Depending on the prostate volume, 2 to 4 laser fibers will be placed bilaterally into the prostate. Patient follow-up consists of uroflowmetry, PROMs, and imaging by using contrast-enhanced ultrasound. Total follow-up is 12 months following treatment.

**Results:**

Presently, recruitment of patients is ongoing. Publication of first results is expected by early 2020.

**Conclusions:**

TPLA offers the potential to be a novel minimal invasive technique for treatment of LUTS due to BPO in men fit for standard desobstruction. This study will evaluate the safety and feasibility of TPLA and report on functional outcomes and tissue changes observed on imaging following TPLA treatment.

**International Registered Report Identifier (IRRID):**

DERR1-10.2196/15687

## Introduction

### Background

Lower urinary tract symptoms (LUTS) in men are mainly caused by benign prostatic hyperplasia. Cross-population prevalence of LUTS in men aged above 40 years is estimated at approximately 20% [1]. The incidence of LUTS due to benign prostatic obstruction (BPO) in the Dutch male population ranges from 3 per 1000 person years at ages 40 to 45 years to 38 per 1000 person years at ages 75 to 79 years [2]. LUTS have a major impact on quality of life compared with other chronic diseases [3,4].

Transurethral surgery is the most common procedure for prostate desobstruction. Numerous techniques have been developed over the years (eg, photo-vaporization and enucleation applying various modalities). The bipolar transurethral resection of the prostate (TURP) has favorable outcomes and a better short-term safety profile compared with monopolar TURP [5]. Photo-vaporization shows equal functional outcomes with a potentially better perioperative safety profile [5]. The holmium laser is the standard for enucleation and is associated with more favorable efficacy outcomes and less complications compared with monopolar TURP [5]. However, all techniques require anesthesia and hospitalization and induce retrograde ejaculation and complications such as hematuria and possible cloth retention while urethral and bladder neck strictures still occur.

Different minimal invasive techniques (eg, interstitial laser coagulation [ILC], transurethral microwave thermotherapy, and transurethral needle ablation) were developed that aimed for equivalent functional outcomes, although with shorter hospitalization, less use of anesthesia, and less complications [6]. Studies comparing these techniques with TURP showed improved patient safety profiles [6-9]. However, long-term functional outcomes were worse when compared with TURP, and the techniques are no longer advised by the guidelines [7,8].

Recently, new techniques (eg, Rezum and Aquablation) were developed. These techniques work with water vapor and waterjet ablation, respectively [10,11]. However, these techniques also use a transurethral approach resulting in similar complications such as hematuria, for example, 3 patients out of 15 for Aquablation [11]. Moreover, Aquablation requires general anesthesia [11]. As these techniques are still being investigated, they are not advised by the guidelines yet. Thus, transurethral surgical treatments are still advised by the guidelines, and there is no percutaneous technique for prostatic desobstruction, which is currently approved [12].

### SoracteLite Transperineal Laser Ablation

SoracteLite transperineal laser ablation (TPLA) of the prostate is a novel minimal invasive technique for LUTS treatment in men. Using a transperineal approach, up to 4 laser fibers can be positioned into the prostate by means of transrectal ultrasound guidance. TPLA generates light-induced thermal heating that results in coagulative necrosis around the laser fiber tip. TPLA requires only local anesthesia, and conscious sedation is optional. Hypothetically, the ablation is confined to the transition zone, respecting anatomical structures and potentially increasing the chance of preservation of antegrade ejaculation. However, as TPLA is a novel technique, only limited short-term outcomes are available. Patelli et al [13] demonstrated technical feasibility and safety of TPLA treatment and improvement of functional outcomes for patients unfit for TURP. Therefore, there is a need to study the safety and feasibility of TPLA treatment of men eligible for standard surgical treatments [13].

### Aim

The aim of this study is to prove safety and feasibility of TPLA treatment for men with BPO eligible for standard surgical treatment.

## Methods

### Study Objectives

The primary objective is to determine safety and feasibility of TPLA treatment for men with LUTS due to BPO and if fit for standard surgical treatment.

Secondary objectives are to determine (1) functional outcomes, (2) the possibility of spontaneous voiding immediately post-TPLA, (3) observation of side effects comprising hematuria, irritative voiding complaints, erectile function, and changes in ejaculation, and (4) evaluation of tissue changes by using contrast-enhanced ultrasound (CEUS).

### Outcomes

Feasibility is measured by technically successfully (without device-related adverse events) performed TPLA procedures. If 90% (18/20) of the treatments are successfully performed, we conclude that the treatment is feasible. Safety is assessed by the adverse events using the CTCAE version 5.0. TPLA is defined to be safe when ≤10% (2/20) of the patients experience adverse events of grade 3 or higher. For the secondary objectives, the functional outcomes are measured by uroflowmetry and International Prostate Symptom Score (IPSS) and erectile function by International Index of Erectile Function 15 (IIEF-15).

### Study Design

This is a prospective, single center, interventional pilot study. Approval of the local medical ethical committee was obtained for the study protocol (registry number: NL66057.018.18). The study is in agreement with the IDEAL stage 2a recommendation [14].

### Population and Sample Size

Patients eligible for this study are men aged ≥40 years with proven bladder outlet obstruction by urodynamic investigation and a peak urinary flow of ≥5 mL per second to ≤15 mL per second, a postvoid residual of ≤250 mL, and a prostate volume of ≥30 and ≤120 cc. The inclusion and exclusion criteria are summarized in Textboxes 1 and 2.

**Inclusion criteria.**

- Male
- ≥40 years of age
- Peak urinary flow: ≥5 mL per second to ≤15 mL per second, minimum voided volume of >125 mL, measured with uroflowmetry
- Postvoid residual: ≤250 mL
- Prostate volume: ≥30 and ≤120 cc, measured by transrectal ultrasound
- Urodynamically proven bladder outlet obstruction

**Exclusion criteria.**

- Previous invasive prostate intervention
- History of prostate or bladder cancer
- No spontaneous voiding (eg, indwelling Foley catheter, clean intermittent catheterization, or suprapubic catheter)
- A clinical suspicion of prostate cancer based on:
  - Abnormal digital rectal examination
  - Urologists’ judgment of the PSA level, preferably supported by a nomogram (eg, Prostaatwijzer version 3 with TRUS volume [16]) outcome with an indication for prostate biopsies
- Inability of temporary discontinuation of anticoagulation or antiplatelet therapy
- Other conditions/status
  - Active urinary tract infection/prostatitis
  - Macroscopic hematuria without a known contributing factor
  - Poor detrusor muscle function or other neurological disorder that would impact bladder function (eg, multiple sclerosis, Parkinson’s disease, spinal cord injuries, (diabetic) polyneuropathy)
  - Concurrent malignancy except basal skin cancer
  - History of pelvic radiation therapy or radical pelvic surgery
  - History of bladder neck contracture and/or urethral strictures within the 5 years before the informed consent date
  - Bladder stones
  - Medical contraindication for undergoing TPLA surgery (eg, infection, coagulopathy, significant cardiac, or other medical risk factors for surgery)
  - Diagnosed or suspected bleeding disorder
  - Contraindication for conscious sedation (eg, obstructive sleep apnea syndrome)

**Figure 1:**
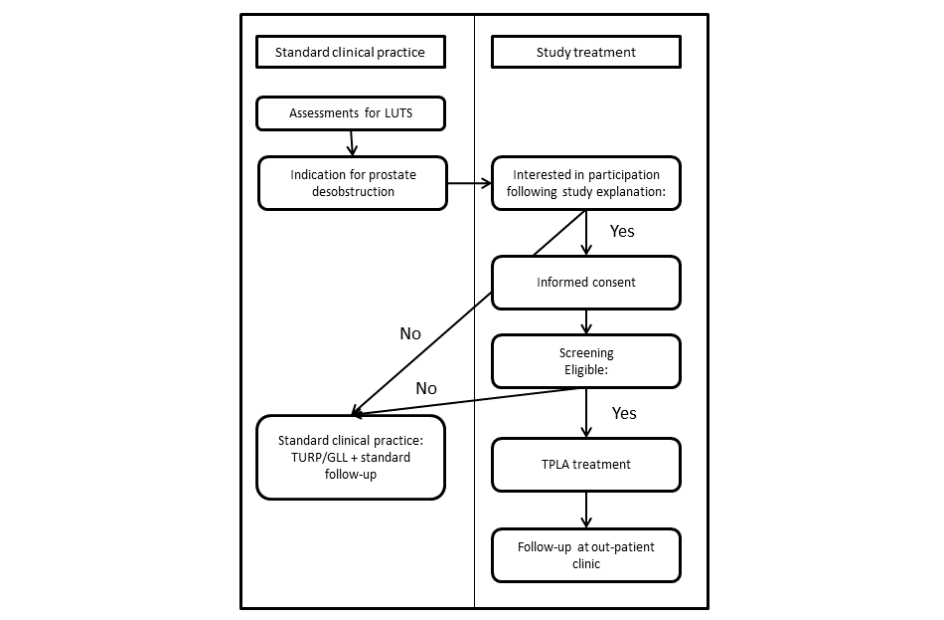
Flow diagram of the study design. LUTS: lower urinary tract symptoms; TPLA: transperineal laser ablation.

All patients will be recruited in the Amsterdam University Medical Centers, Academic Medical Center (Amsterdam, the Netherlands). Patients will be informed about this study in oral and written form. Patient approval is confirmed by signed informed consent. Patient inclusion has been summarized in a flow diagram (Figure 1).

Overall, 20 patients will be included in this study. This number is based on the IDEAL stage 2a recommendations for surgical innovations and past pilot studies [13-15].

### Baseline Characteristics

Medical history and physical examination will be evaluated. In addition, IPSS and IIEF-15 will be used to measure voiding and erectile symptoms. Pain is measured by using a visual analogue scale (VAS), ranging from 0 to 10, and hematuria by the hematuria grading scale (HGS) [17]. Voiding characteristics and bladder function will be assessed by uroflowmetry with postvoid residual measurement and urodynamic investigation. Prostate volume measurement will be performed by transrectal ultrasound.

### Study Device

**Figure 2:**
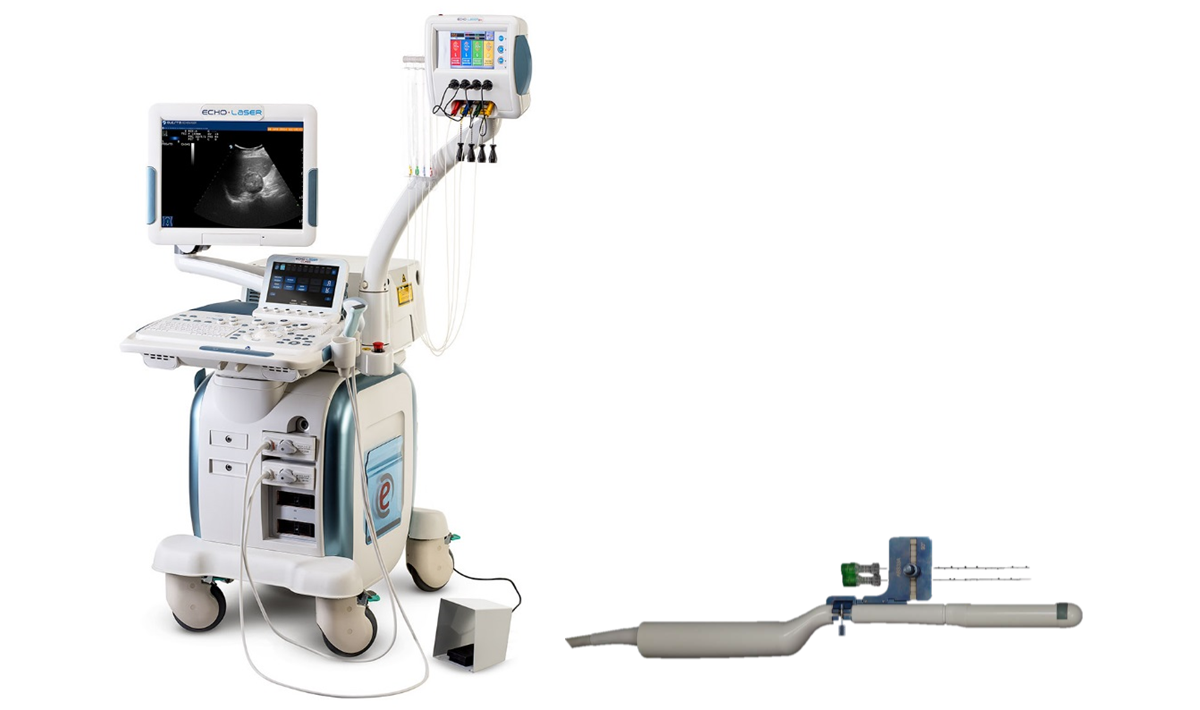
The EchoLaser system with 4 fibers combined with the MyLab Eight eXP ultrasound device from Esaote (left) and the TRT33 probe with needle guide system (right).

The TPLA treatment is performed using a diode laser system (SoracteLite, EchoLaser X4 system, Elesta; Figure 2). The system uses 4 diode laser sources, operating at 1064-nm wavelength, with a maximum energy of 7 watts continuous wave per source. The laser sources can be activated and configured individually (both separately and simultaneous), and the laser light is guided through a flexible quarts optical fiber.

The laser light induces tissue coagulation with a relatively deep penetration (up to 1 cm) because light of 1064 nm is poorly absorbed by hemoglobin and water [18]. However, fiber blood contact can lead to carbonization of the fiber tip, creating a black layer that partially absorbs the light [19]. Subsequently, the absorbed light is transformed into heat because of the black layer and other tissue constituents in the prostate. During TPLA, the prostate is continuously monitored by using transrectal ultrasound (MyLab Eight eXP, Esaote) with a biplanar probe (TRT33).

### Study Procedure

**Figure 3:**
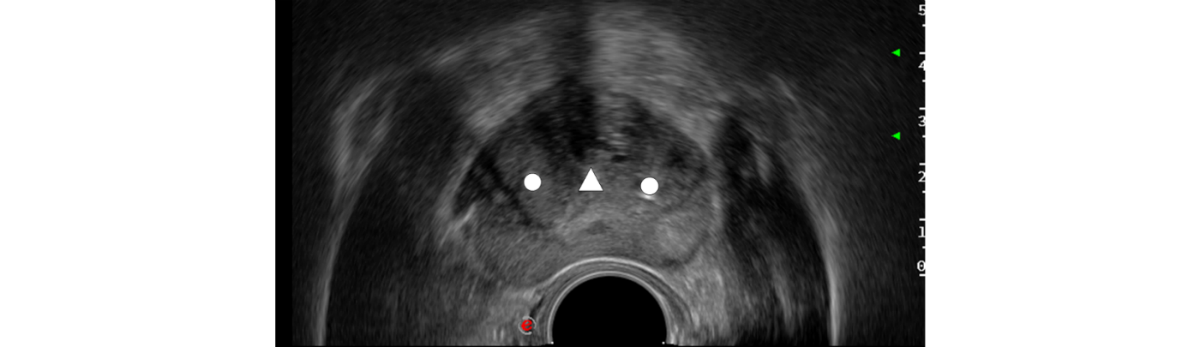
Transverse plane ultrasound image of the prostate. Fibres (circles) positioned in both transition zones while maintaining a safe distance from the urethra (triangle), rectum and prostatic capsule.

The TPLA treatment will be performed by 2 urologists who followed a training to perform the TPLA procedure. TPLA treatment will be performed in an outpatient setting under local anesthesia. A single oral dose of ciprofloxacin 500 mg will be used as antibiotic prophylaxis. The patient will be placed in lithotomy position. A Foley catheter will be placed for urethral identification. The prostate will be visualized with a biplanar transrectal ultrasound probe. Pain management consists of local infiltrative anesthesia of the perineum with Lidocaine 2%, 5 to 10 mL, followed by an ultrasound-guided periprostatic block with Lidocaine 2%, 20 mL. On patients’ preference, conscious IV sedation with Midazolam 1 to 3 mg is offered. Depending on the prostate volume, 1 or 2 fibers will be placed transperineally in each side of the prostate in the transition zone by using ultrasound guidance and guiding needles (Figure 3 and Table 1). The fibers will be positioned parallel to the urethra while maintaining a minimal distance of 8 to 10 mm to the urethra and the rectal wall, and a distance of at least 15 mm to the bladder neck. A needle guide system will be used to support parallel placement of the needles. Following introduction of the optical fiber through the needle, the needle will be retracted for 10 mm, allowing exposure of the fiber tip and first 10 mm of the fiber to the tissue. When 2 fibers are located within the same lobe, the distance between the needles will be 10 or 15 mm, depending on the prostate size. If a median lobe is present, it will be treated by placing a needle and fiber as well.

Ablation will be performed with 3 watts per fiber and a total energy of 1800 J will be delivered per fiber in 600 seconds. Depending on the prostate volume, a pullback can be performed, retracting the fiber 1 cm along its trajectory to deliver another 1800 J (Table 1). When the treatment is completed, the Foley catheter will be removed. Dexamethasone 8 mg is administered intravenously postoperative to reduce edema and will be continued orally with the same dosage for 7 days.

**Table 1:** Overview of the number of fibers used and fiber distance based on prostate volume (based on the manufacturer’s instructions).

Prostate volume (cc)	Power (watts)	Energy per fiber (J)	Number of fibers per lobe	Fiber distance (mm)	Number of pullbacks	Total delivered energy (J)
30-40^a^	3	1800	1	—^b^	0	3600
	3	1800	1	—	1	7200
40-80^a^	3	1800	1	—	1	7200
	3	1800	2	10-15	0	7200
	3	1800	2	10-15	1	14,400
>80^a^	3	1800	2	10-15	1	14,400
	3	1800	2	10-15	2	21,600

^a^The operator, based on the prostate shape and length, should determine the optimal fiber setup.

^b^Not applicable.

Following the procedure, the patient will be observed for several hours for spontaneous voiding without significant residual urine. If the patient does not have spontaneous voiding or has a residual volume of ≥500 mL, an indwelling catheter will be placed. For these cases, 10 to 14 days later, a trial without a catheter will follow.

### Contrast-Enhanced Ultrasound

**Figure 4:**
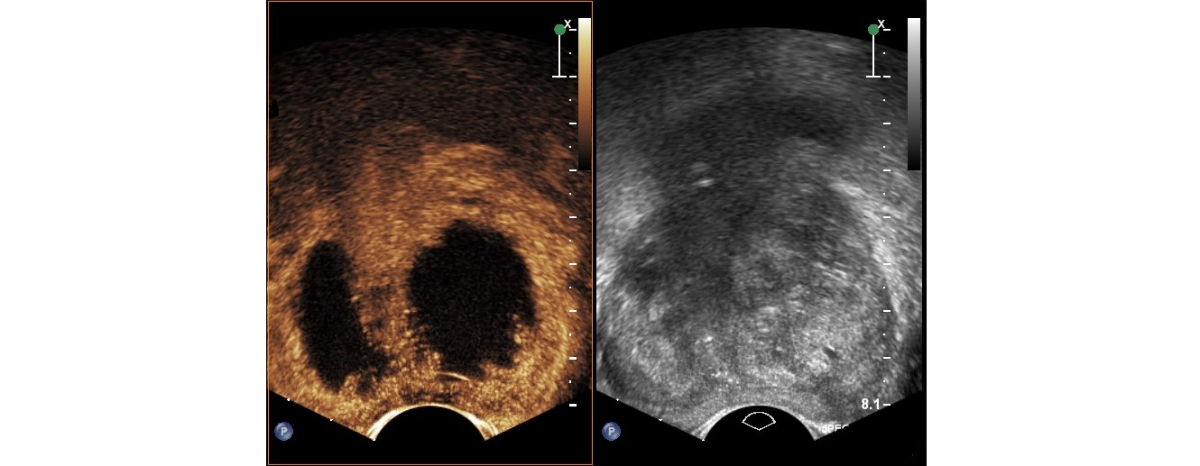
On the left the contract-enhanced ultrasound image of the prostate of the prostate 1 month following TPLA treatment, with dark areas corresponding with the treated areas. On the right the co-localized grey-scale image.

Multiple CEUS investigations of the prostate will be performed over the course of this study. CEUS is used because the ablation zone is not visible on gray-scale ultrasound (Figure 4) [20]. The contrast agent is a gas-filled microbubble (Sonovue, Bracco). This contrast agent enables visualization of the vascular architecture of the prostate. Thus, nonvascularized coagulated areas are visible as hypo-intense (black) regions [21]. Several studies have used CEUS for laser ablation area visualization in prostate cancer.

Lindner et al demonstrated that CEUS shows a clear hypo-intense zone, which corresponds with the ablated area following focal laser ablation of prostate cancer [21]. Patelli et al confirmed this result by demonstrating the applicability of CEUS for ablation zone determination following TPLA treatment [13]. CEUS is considered safe, because the lungs eliminate the contrast agent and ionizing radiation is not needed. A hypersensitivity allergic reaction to the contrast agent is a serious, but rare adverse event (<0.01%) [22].

CEUS imaging will be performed according to internal hospital protocol, with a Philips iU 22 machine interfaced with an end-firing transrectal probe (Philips Healthcare, Bothell). Prostate imaging consists of prostate volume measurement in 2 directions in B-mode (gray scale) setting. This will be followed by a transverse and sagittal sweep of the prostate in contrast mode, during peak inflow of the first bolus of 2.4 mL contrast agent. Subsequently, images will be made of the maximal diameter of both lesions in the sagittal and transverse planes. Treatment volumes will be calculated on the contrast images using an ellipsoid formula [23]. The second bolus will be used to capture contrast inflow and outflow within 1 plane over the course of 2 min.

### Follow-Up

Patients will undergo a strict follow-up. The patient will be called 3 to 5 days post-TPLA treatment to inquire about health status. The patient will visit the hospital 4 times following TPLA treatment at 4 weeks, 3 months, 6 months, and 12 months. During these visits, medical history, adverse events using the Clavien-Dindo classification, physical examination (on indication), and uroflowmetry will be performed. Patient-reported outcome measures (PROMs) (IPSS, IIEF-15, VAS, and HGS) are used to objectify functional outcomes, pain, and symptoms. Prostate imaging will comprise a CEUS +/– 2 hours following treatment, at 4 weeks and 12 months. CEUS is considered the only invasive procedure during follow-up. Follow-up is completed after 12 months.

### Data Analysis

Study population characteristics (eg, age, medical history, medication, peak urinary flow rate, prostate volume, and detrusor pressure) and procedural information (eg, fibers placed, energy delivered, procedural success, adverse events, and lesion volume) will be reported descriptively. Functional outcomes by peak urinary flow and PROMs will be tested using a Wilcoxon signed-rank test. Statistical tests will be performed two-sided, and *P*<.05 is considered significant. Statistical analysis will be performed using SPSS version 25.0 (SPSS Inc).

### Safety

The investigators will monitor patient safety. They can withdraw a patient from the study for medical reasons. In accordance to section 10, subsection 4, of the Wet Medisch-Wetenschappelijk Onderzoek met Mensen (medical research involving human subjects act in the Netherlands), the investigators will suspend the study if there is sufficient ground that continuation of the study will jeopardize patients’ health or safety. The investigators will notify the accredited institutional review board if this is the case. In case of an adverse event or serious adverse event, the responsible authorities will be informed.

### Benefits and Risks

All patients included in this study have LUTS and have an indication for prostate desobstruction. This study aims to treat patients for their LUTS by TPLA to evaluate safety and feasibility and functional outcomes. The benefits focus on the minimal invasive character of TPLA and subsequent day care setting without clinical admission. Spinal or general anesthesia is made redundant by use of local anesthesia and optional conscious sedation.

Risks for patients focus on the procedure, subsequent device-related adverse events, and unknown long-term functional outcomes of the treatment. Needles are used to introduce fibers into the prostate, possibly causing damage and perforation of surrounding structures (eg, neurovascular bundle, urethra, or rectal wall). As ultrasound guidance is being used, these events are unlikely to occur. Hemorrhage, infection, fistula formation, sepsis, and death can occur. However, risks are expected to be low because the transperineal approach is already applied for biopsies of the prostate and has proven to be safe [24]. Furthermore, Patelli et al reported no procedure-related adverse events in 18 patients [13]. Therefore, risks of adverse events are minimal as described earlier.

Nonetheless, TPLA is a relatively new technique and long-term outcomes are unknown. Patelli et al described short-term functional outcomes demonstrating that the peak urinary flow rate improved from 7.6 to 13.3 mL per second at 3 months post-TPLA. Yet, studies that applied other minimal invasive LUTS treatments showed a reduction of long-term effectiveness and increase of retreatment rates over time [8,9]. Thus, when feasibility of TPLA in patients that are candidates for surgical desobstruction can be demonstrated, future studies will be able to focus on the long-term outcomes.

## Results

Presently, patient recruitment is ongoing. Initial results on safety and feasibility are expected in early 2020. Follow-up outcomes following TPLA treatment are expected in late 2020. Outcomes will be published in peer-reviewed medical journals and presented at international conferences.

## Discussion

### Overview

TPLA is a technique that might efficiently treat BPO under local anesthesia in an outpatient setting. This pilot study will provide initial data on the safety and feasibility of TPLA treatment in healthy men eligible for standard surgery before launching large-scale comparative studies.

### Comparison With the Literature

The design of our study aims to show safety and feasibility in men fit for standard surgical treatment. The first TPLA pilot study by Patelli et al [13] showed promising results for men unfit for standard treatment. They treated 18 patients and showed technical safety and feasibility for men unfit for TURP due to comorbidities [13]. Functional outcomes showed peak urinary flow improvement from 7.6 (SD 2.7) to 13.3 (SD 76.2) mL per second at 3 months. IPSS improved from 21.9 (SD 6.2) to 10.7 (SD 4.7) at 3 months. Interestingly, a relatively long postoperative catheterization time of 17.3 (SD 10.0) days was observed. However, half of the study population already needed an indwelling catheter before TPLA treatment. Our study aims to study the safety and feasibility of TPLA treatment for men fit for standard treatment. Our study also aims to confirm the outcomes of previous studies, in a population with proven bladder outlet obstruction. Additionally, our study aims to preserve spontaneous voiding posttreatment by including men with spontaneous voiding before treatment. Nevertheless, risk for obstruction following treatment due to edema remains. Therefore, outcomes regarding spontaneous voiding post-treatment are evaluated after 10 patients have been treated. If more than 50% of treated patients do not spontaneously void, the subsequent 10 patients will receive a Foley catheter for 10 to 14 days.

### Transperineal Laser Ablation Versus Interstitial Laser Coagulation

Interestingly, TPLA is based on the technique of inducing coagulative tissue necrosis, which is similar to the tissue response found in transurethral ILC. However, the long-term outcomes of ILC appeared to be inferior to TURP [7]. As ILC and TPLA are based on a similar concept, it could cause criticism studying this approach. However, the currently available TPLA treatment has benefits when compared with the previous ILC treatment.

First, TPLA uses a transperineal approach in contrast to ILC which uses a transurethral fiber introduction. The transperineal approach of TPLA treatment aims to leave the urethra undamaged, hereby reducing post-treatment irritative voiding complaints when compared with the transurethral approach, which causes urothelial damage and subsequent irritative voiding complaints.

Second, the multifiber setup of TPLA treatment enables simultaneous treatment of both prostate lobes with 1 or 2 fibers each depending on prostate volume. If a median lobe is present, this can be treated as well. Additionally, each fiber can be configured individually, which enables shaping of the ablation zone. Hereby, a desired ablation volume is obtained faster when compared with serial treatment with 1 fiber used with ILC. Thus, the unique multifiber approach is expected to increase ablation volume faster and subsequently improved outcomes, especially in larger prostates.

Finally, TPLA is performed under continuous ultrasound imaging guidance, which provides visualization of the complete gland. This leads to improved treatment planning and ablation zone shaping, and subsequently theoretically improved outcomes.

### Study Limitations

Nonetheless, this pilot study has several limitations. First, inclusion is set at 20 patients and is considered sufficient for determining safety and feasibility. However, this is not sufficient for measuring functional outcomes, as the study is not powered for this. Thus, successive studies are necessary once this study shows initial beneficial results. In addition to this, we have initiated an international registry to collect data from other centers that apply TPLA for BPO (ClinicalTrial.gov registration: NCT03776006). Second, a learning curve might be expected in the number of fibers placed and their location. Therefore, only a limited number of physicians will perform the TPLA procedures for this pilot.

### Conclusion

In conclusion, TPLA treatment offers a unique multifiber and transperineal approach for the treatment of LUTS due to BPO under local anesthesia resulting in ablation zone shaping and creation of an ablation zone volume in a short period of time. We hypothesize that this study will confirm safety and feasibility of TPLA treatment of men with LUTS due to BPO and fit for standard surgery. The results of this study will broaden the knowledge on TPLA treatment in men with LUTS and are expected to be an essential basis for future BPO treatment using this approach.

## Abbreviations

BPO: benign prostatic obstruction
CEUS: contrast-enhanced ultrasound
HGS: Hematuria Grading Scale
IIEF: International Index of Erectile Function
ILC: interstitial laser coagulation
IPSS: International Prostate Symptom Score
LUTS: lower urinary tract symptoms
PROM: patient-reported outcome measure
TPLA: soracteLite transperineal laser ablation
TURP: transurethral resection of the prostate
VAS: visual analogue scale
